# Effects of rhomboid intercostal and sub-serratus plane block on perioperative analgesic efficacy and diaphragm excursion in video-assisted thoracic surgery: a prospective, randomized controlled trial

**DOI:** 10.1186/s12871-025-03556-3

**Published:** 2025-12-18

**Authors:** Shi-Fei Zhao, Quan-Yuan Chang, Ya-Ru Zheng, Lan Qiu, Jiang Shen

**Affiliations:** 1 Department of Anesthesiology, The Third Affiliated Hospital of Soochow University, Changzhou, Jiangsu 213000 China; 2 Department of Anesthesiology, Changzhou First People’s Hospital, Changzhou, Jiangsu 213000 China; 3 Department of Anesthesiology, Changzhou Fourth People’s Hospital, Changzhou, Jiangsu 213000 China

**Keywords:** Video-assisted thoracic surgery, Rhomboid intercostal and sub-serratus plane block, Diaphragmatic excursion, Postoperative analgesia

## Abstract

**Background:**

Rhomboid intercostal and sub-serratus plane (RISS) block is a novel nerve block technique that provides good analgesia, but overall research is scarce. This study aimed to investigate the effect of ultrasound-guided RISS block on postoperative analgesia and diaphragmatic excursion (DE) after video-assisted thoracic surgery (VATS) for lung cancer.

**Methods:**

One hundred patients who underwent VATS lung resection participated in this study and were randomized to a RISS group (Group R) or a control group (Group C). Group R underwent ultrasound-guided RISS block with 0.25% ropivacaine hydrochloride 0.5 ml/kg immediately after surgery. Group C was given standard general anaesthesia, and patient-controlled intravenous analgesia (PCIA) was used in the postoperative period in both groups. The visual analogue scores (VAS) at rest and during movement at 2 hours(h), 24 h, and 48 h postoperatively were used as the primary outcome measures. Secondary outcomes included postoperative consumption of sufentanil; preoperative and postoperative left and right-sided DE during calm and deep breathing, and the occurrence of adverse effects such as postoperative nausea and vomiting (PONV), dizziness, somnolence, puncture site infection, and hematoma.

**Results:**

At 2 h, 24 h, and 48 h postoperatively, patients in Group R had lower VAS scores at rest (median [Q1, Q3]: 1.00 [1.00, 1.00]; 1.00 [1.00, 1.00]; 0.00 [0.00, 1.00]) and during movement (2.00 [2.00, 3.00]; 2.00 [2.00, 2.00]; 2.00 [1.00, 2.00]) than those in Group C (resting: 2.00 [2.00, 2.00]; 2.00 [2.00, 2.00]; 1.00 [1.00, 2.00]; movement: 3.00 [3.00, 4.00]; 3.00 [3.00, 4.00]; 3.00 [2.00, 4.00]) (all *P* < 0.0001). Sufentanil consumption at 2 h, 24 h, and 48 h postoperatively was also significantly lower in Group R (*P* = 0.0002, *P* < 0.0001, *P* < 0.0001). Preoperatively, no significant difference in DE existed between the groups (*P* > 0.05). At 30 min post-extubation and 2 h and 24 h postoperatively, during both calm and deep breathing on both sides, Group R had significantly greater DE than Group C (*P* < 0.05). At 48 h postoperatively, the right-sided DE during calm breathing showed no significant difference between the groups. PONV incidences did not differ significantly (*P* = 0.2662), and Group R had less dizziness, somnolence, higher satisfaction post-surgery, and showed no cases of puncture site infection or hematoma.

**Conclusions:**

Ultrasound-guided RISS block can modestly reduce postoperative pain in patients undergoing VATS, with clinically relevant benefits, and may help alleviate diaphragmatic dysfunction caused by surgical or anesthetic factors.

**Trial registration:**

The trial was registered at the China Clinical Trial Registry (http://www.chictr.org.cn, ChiCTR2300070842) on 24/04/2023.

**Supplementary Information:**

The online version contains supplementary material available at 10.1186/s12871-025-03556-3.

## Introduction

Video-assisted thoracic surgery (VATS) has been widely used for lung cancer treatment. Despite its smaller incisions and less surgical trauma than traditional open thoracic surgery, 50%−70% of patients still experience moderate-to-severe postoperative pain [1]. After lung cancer resection, long-term persistent pain makes patients avoid coughing and expectoration, leading to secretion retention and subsequent postoperative atelectasis, pneumonia, etc., severely affecting patients’ quality of life and rehabilitation. Therefore, perioperative analgesia is crucial. Domestic and international guidelines recommend multimodal analgesia for thoracic surgery perioperatively [2], and the regional nerve block-plus-patient-controlled intravenous analgesia (PCIA) postoperative analgesia regimen is widely used in clinical anesthesia [2].

Rhomboid intercostal and sub-serratus plane block (RISS), as an emerging nerve block technique proposed by H. Elsharkawy et al. in 2016 [3], involves the precise ultrasound-guided injection of local anesthetic drugs into the fascial layer between the rhomboid and intercostal muscles, as well as between the anterior serratus and intercostal muscles in the 5th and 6th intercostal spaces. This technique can effectively block the surrounding nerve conduction and reduce the afferent transmission of harmful stimuli, achieving a good analgesic effect. Although RISS block has been initially applied in clinical practice, and some study results have shown that it has demonstrated an excellent perioperative analgesic effect for a wide range of surgical procedures, overall research is scarce, and more studies are required to further evaluate its safety and efficacy.

Respiratory function recovery is crucial in assessing postoperative recovery in thoracic surgery [4]. The diaphragm is a key respiratory muscle, providing about 2/3 of lung ventilation during quiet breathing. Diaphragm excursion (DE), the displacement between end-inspiration and end-expiration, is a vital index for assessing diaphragm and respiratory recovery [5]. In both traditional open-heart surgery and VATS [6], factors like general anesthesia and mechanical ventilation can reduce diaphragm movement amplitude [7, 8], affecting its function and leading to complications such as hypoventilation syndrome and atelectasis [9, 10]. Thus, timely diaphragm function assessment is highly significant for patients. Diaphragm ultrasonography is important for diagnosing respiratory and thoraco-abdominal diseases. Compared to X-ray fluoroscopy, it is non-invasive, portable, and radiation-free, with high sensitivity (93%) and specificity (100%) in diagnosing diaphragm dysfunction. It enables full-process diaphragm function tracking and timely detection of perioperative diaphragm paralysis and impairment [7, 11]. Consequently, ultrasound-guided DE examination can be used to evaluate diaphragm and respiratory function recovery.

This study aims to verify that RISS block can provide satisfactory peri-operative analgesia for VATS patients and has a protective effect on patients’ respiratory function by observing the peri-operative analgesic effect of VATS patients with RISS block technique and measuring DE at different peri-operative time points, to further accelerate patients’ postoperative rehabilitation and improve their quality of life.

## Patients and methods

### Research objects

This single-center, prospective, randomized controlled study received ethical approval from the Ethics Committee of the Third Affiliated Hospital of Soochow University, Approval number ((2023) Section No. 033), and written informed consent was obtained from all patients. We registered the study protocol with the China Clinical Trial Registry (http://www.chictr.org.cn, ChiCTR2300070842) on April 24, 2023. The study design adhered to the CONSORT guidelines. The study protocol and study process strictly followed the Declaration of Helsinki and the requirements of the Ethics Committee. One hundred patients who underwent VATS lung resection under general anesthesia in the Third Affiliated Hospital of Soochow University from May 1 to July 31, 2023 were recruited. One hundred patients enrolled in this study met the following inclusion criteria: (1) age: 20–80 years old; (2) ASA (American Society of Anesthesiologists) classification: I - III; (3) patients undergoing elective thoracoscopic lung cancer surgery. Exclusion criteria were as follows: (1) abnormal blood coagulation function; (2) infection at the puncture site; (3) immune system or endocrine dysfunction; (4) allergy to opioids, non-steroidal anti-inflammatory drugs, hormones or other immunomodulatory substances.

### Grouping and blinding method

A random number Tables (1–100) was generated using SPSS 27.0 to allocate patients randomly into two groups in a 1:1 ratio. Group R received general anesthesia combined with a RISS block, while Group C received general anesthesia alone. Both groups received PCIA. Group allocation was concealed in sequentially numbered, opaque, sealed envelopes. On the day of surgery, an anesthesia nurse not involved in the study opened the sealed envelope. If the patient was assigned to Group R, the nurse informed a designated experienced anesthesiologist (also not involved in the trial) to perform DE measurement and administer the RISS block. For patients assigned to Group C, the same anesthesiologist performed DE measurement but did not perform the RISS block. Postoperative follow-up and outcome assessments were conducted by another anesthesiologist who was blinded to group allocation. Patients in Group R received the RISS block while still under general anesthesia, and thus were unaware of their group assignment. Therefore, both patients and outcome assessors remained blinded to the group allocation.

### DE measurement

**Fig. 1:**
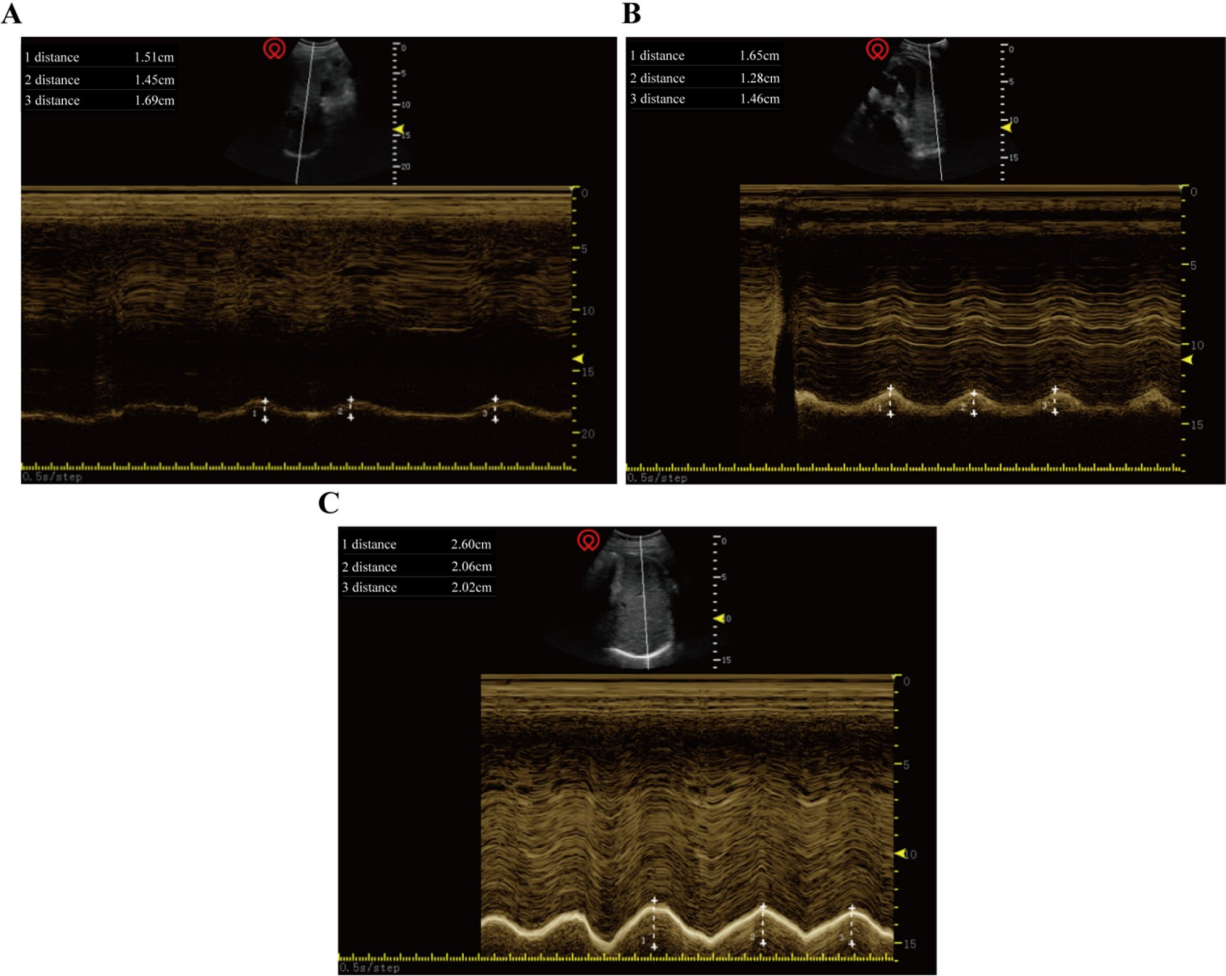
Ultrasound images of the left and right diaphragms in M-mode and measurement of diaphragmatic excursion. **A** Ultrasound image of the right-side diaphragm (liver acoustic window); **B** Ultrasound image of the right-side diaphragm (Splenic acoustic window); **C** Measurement of diaphragmatic excursion under M-mode ultrasound. Note: When three consecutive stable waveforms appear in the DE waveform shown by M ultrasound, the distance from the baseline to the crest of the waveform is measured and the average value (shown as 1, 2, and 3 in the figure) is recorded as the DE

All measurements were completed by the same experienced anesthesiologist. The patient was placed in a semi-recumbent position with the head of the bed elevated 30°; a low-frequency convex-array ultrasound probe of 2–5 MHz was selected; when the patient was breathing calmly, the ultrasound probe was placed in the B-mode ultrasound mode directly below the intersection of the midclavicular line and the lower edge of the rib arch, and the ultrasound window was taken as the liver on the right side [12](Fig. 1A) and the spleen on the left side [13](Fig. 1B). A bright echo line was observed on the diaphragm ultrasound image, which moved with respiration; then the ultrasound beam was made perpendicular to the posterior 1/3 of the diaphragm, converted to M-mode ultrasound, and the diaphragm movement trajectory was measured as a sinusoidal curve, and the distance from the baseline on the vertical axis to the highest point in inspiration was measured, which was the DE, the average of the DE for three consecutive times was taken (Fig. 1C**)**. Preoperatively, 30 min after extubation, 2 h(h), 24 h, and 48 h after the operation, bedside bilateral ultrasound-guided DE measurements were carried out respectively and their measured values were recorded.

### Anesthesia procedure and postoperative analgesia

Standard anesthesia monitoring, including continuous electrocardiogram, pulse oximetry, electroencephalographic bispectral index (BIS), and non-invasive blood pressure, was initiated after the patient entered the operating room. After verification, all patients received pre-oxygenation, and anesthesia was induced with etomidate 0.2–0.3 mg/kg, sufentanil 0.3-0.3.5ug/kg, propofol 2–3 mg/kg, and cisatracurium besylate 0.2–0.3 mg/kg, and after the patient’s consciousness completely disappeared, double - lumen endotracheal intubation was performed under the guidance of a video laryngoscope, and the patient was placed in the lateral decubitus position. General anesthesia was maintained by inhalation of sevoflurane (about 1 MAC), intravenous infusion of propofol (3–6 mg·kg⁻¹·h⁻¹) and remifentanil (0.5–1.0 µg·kg⁻¹·h⁻¹), with the fluctuation of hemodynamic parameters maintained within 20% of the baseline, and the BIS was maintained at 40–60. Muscle relaxation was achieved by intermittent injections of cisatracurium besylate as needed. Both groups were routinely administered 5–10 µg of sufentanil and 50 mg of flurbiprofen axetil for analgesia before skin closure.

All surgeries were performed by the same thoracic surgical team. A three-port approach was used: the main surgical port was located at the anterior axillary line between the 4th and 5th ribs, the thoracoscopic port at the mid-axillary line between the 6th and 7th ribs, and the assistant port at the posterior axillary line between the 8th and 9th ribs. A single chest drainage tube was placed at the mid-axillary line between the 6th and 7th ribs.

Both groups treated with the same postoperative multimodal analgesic management protocol. all patients routinely received 5–10 µg of sufentanil and 50 mg of flurbiprofen axetil for analgesia prior to skin closure. Postoperatively, a PCIA pump was used, containing sufentanil 2 µg/kg and esketamine 0.3 mg/kg, diluted to a total volume of 100 mL. The background infusion rate was set at 2 mL/h, with a bolus dose of 2 mL and a lockout interval of 15 min. In the ward, when the static VAS ≥ 4 or there was a need for analgesia, patients were instructed to press the PCIA pump once. However, If analgesia remained inadequate, intravenous flurbiprofen axetil 50 mg was administered as rescue analgesia, with a maximum daily dose not exceeding 200 mg.

### RISS block

**Fig. 2:**
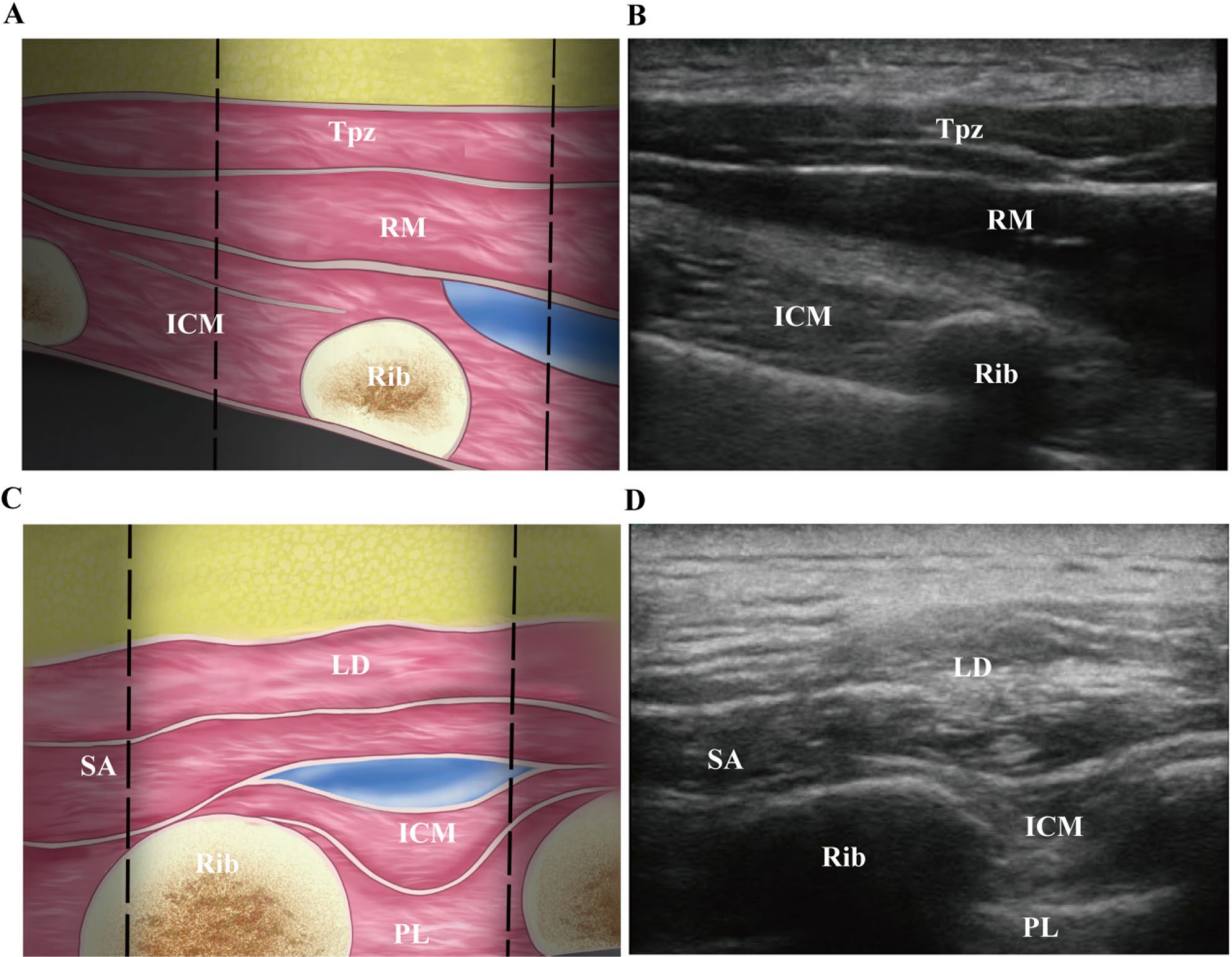
Schematic diagrams and ultrasound images of the RISS block. **A** Schematic diagram of rhomboid-intercostal plane block; **B** Ultrasound image of rhomboid-intercostal plane block; **C** Schematic diagram of low serratus anterior plane block; **D** Ultrasound image of low serratus anterior plane block. Tpz: Trapezius muscle; RM: Rhomboid muscle; Rib: Rib; ICM: Intercostal muscle; LD: Latissimus dorsi; SA: Serratus anterior; PL: Pleura; Blue color: Drug solution

All nerve block operations were completed by the same experienced anesthesiologist. In Group R, the RISS block was immediately performed after the operation ended. The patient maintained the same lateral decubitus position as during the operation, and the side of the operation was the block side. A high-frequency linear-array probe (Navi T-type, 8–14 MHz, Huasheng color ultrasound diagnostic system) was selected, and a low-frequency convex-array probe (2–6 MHz) could be used for obese patients. Routine disinfection and draping were carried out, and the ultrasound probe was wrapped with a sterile sleeve. The block was performed in two steps:

1) Rhomboid Intercostal Block (RIB): The ultrasound probe was first placed in the coronal plane along the medial border of the scapula at the T5–T6 level on the surgical side. It was then rotated 90 degrees into the sagittal plane along the inferior medial border of the scapula, with the probe marker directed cephalad. The ultrasound image displayed tissue structures in the following order: trapezius, rhomboid major, intercostal muscles, ribs, and pleura. An in-plane needle insertion was performed in the cephalocaudal direction. The needle passed sequentially through the trapezius and rhomboid major muscles and advanced into the fascial plane between the rhomboid major and intercostal muscles. After confirming correct needle tip placement using 5 mL of normal saline for hydrodissection and ensuring negative aspiration for blood or air, half the total dose of 0.25% ropivacaine (0.5 mL/kg) was injected. The local anesthetic spread in a spindle-shaped pattern within the deep serratus anterior fascial plane, diffusing cephalad, caudally, and laterally to block the lateral cutaneous branches of the T_6_–T_12_ intercostal nerves. (Fig. 2A-B).

2) Low Serratus Anterior Plane Block (SAPB): The ultrasound probe was then moved caudally and laterally to the T_7_-T_8_ level, posterior to the posterior axillary line and below the inferior angle of the scapula. The sonographic anatomy included the latissimus dorsi, serratus anterior, intercostal muscles, pleura, and lung. The needle was advanced in a caudolateral direction. Once the tip reached the fascial plane between the serratus anterior and intercostal muscles, and after confirming negative aspiration, 5 mL of saline was injected to verify proper needle position. The remaining half dose of 0.25% ropivacaine was then administered. The local anesthetic was observed to spread in a spindle shape within the deep serratus anterior plane, diffusing cephalad, caudally, and laterally to block the lateral cutaneous branches, and medially to the deep surface of the erector spinae muscles, targeting the cutaneous branches of the T_6_–T_12_ spinal nerves (Fig. 2C-D). After the block was completed and the patient had fully regained consciousness, meeting the discharge criteria from the Post-Anesthesia Care Unit (PACU), the anesthesiologist who performed the nerve block assessed the block range using the cold stimulation method. The extent of the block was determined by measuring the sensory area in both the vertical and horizontal directions from the puncture site. If the sensory level at the mid-axillary line was less than two dermatomes, the block was considered a failure and the patient was dropped from the study.

### Outcome measurement

The primary outcome was the visual analog scale (VAS) for pain at rest and during movement at 2 h, 24 h, and 48 h postoperatively. Secondary outcomes included sufentanil consumption at 2 h, 24 h, and 48 h postoperatively; ultrasound measurements of bilateral DE during quiet and deep breathing were recorded preoperatively, at 30 min after extubation, and 2 h, 24 h, and 48 h postoperatively; postoperative adverse effects including postoperative nausea and vomiting (PONV), dizziness, and somnolence; and patient satisfaction. Patient satisfaction was measured using a 4-point Likert scale (0 = dissatisfied; 1 point = basically satisfied; 2 points = relatively satisfied; 3 = very satisfied) [14].

### Sample size and statistical analysis

The sample size was calculated based on the results of the pre-test of 6 patients, which used the VAS score at 24 h postoperatively as the main evaluation index, and the mean value of the VAS score during movement at 24 h postoperatively in the pre-test was 1.8 ± 0.56 in Group R and 3.2 ± 0.58 in Group C, yielding an expected mean difference of 1.4 points. This difference was considered clinically meaningful according to previous studies on postoperative pain management after thoracic surgery, in which a reduction of ≥ 1.0 point on the VAS scale is regarded as clinically relevant. The “pwr” package in R language was used to calculate the required sample size. The difference between the mean/standard deviation of the VAS score in the pre-treatment group and the control group was calculated. The significance level α was set as bilateral 0.05, and the test power 1-β was set at 0.9. After the calculation, the sample size in each group should be no less than 37 cases. With the loss-to-follow-up rate set at 20%, at least 89 samples were required. This study planned to enroll 100 patients to meet the sample size requirement. R language 4.3.1 statistical software was used for data analysis. For measurement data that followed a normal distribution, they were expressed as mean ± standard deviation (`x ± s), and the t-test was used for comparison between groups; for measurement data that conformed to a skewed distribution, non-parametric tests were used and expressed as median (interquartile range) [M (Q1, Q3)]; for count data, they were expressed as frequency n (%) and the chi-square test or Fisher’s exact test was used for comparison between groups. Bonferroni correction was used for multiple comparisons. *P* < 0.05 was considered a statistically significant difference.

## Results

### Patient demographics and operation characteristics

**Fig. 3:**
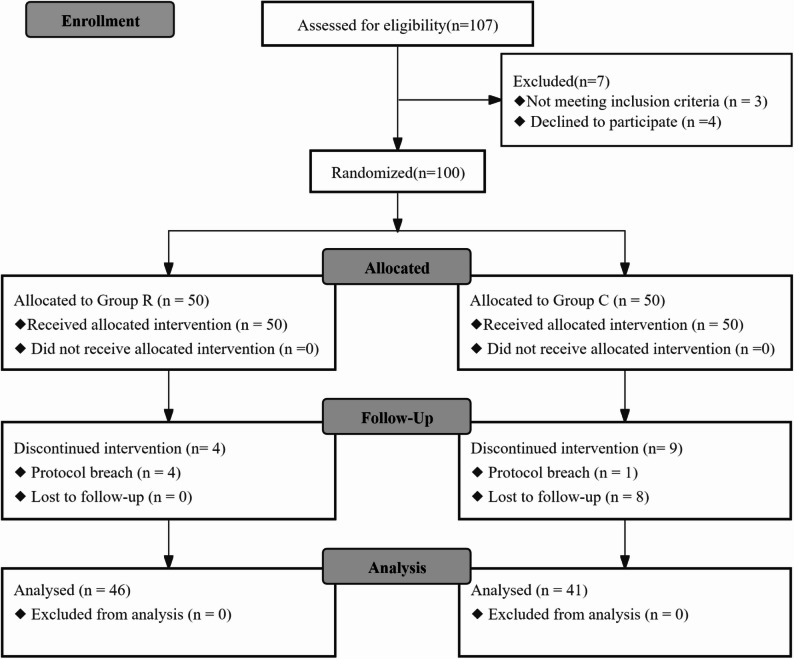
Patient Consolidated Standards of Reporting Trials (CONSORT) flow diagram

According to the CONSORT 2010 statement [15], the flowchart is detailed in Fig. 3. This study included 100 patients who underwent elective VATS lung resection at the Third Affiliated Hospital of Soochow University between May 1, 2023 and July 31, 2023, including 13 patients who withdrew midway (3 patients experienced block failure, 1 patient were delayed postoperative extubation, 1 patient were converted to thoracotomy, and 8 patients were lost during follow-up). The final statistics included 87 patients, 46 in Group R and 41 in Group C. There were no significant differences in gender, age, height, weight, body mass index (BMI), ASA classification, one-lung ventilation time, intraoperative blood loss, surgical approach (left or right side) and intraoperative drug dosage between the two groups (*P* > 0.05, Table 1**)**.

**Table 1:** Patients’ demographic and clinical data

		Group R (*n* = 46)	Group C (*n* = 41)	*P*-value
Gender (n, %)	male	20 (43.48)	16 (36.59)	0.6632
	female	25 (56.52)	26 (63.41)	0.6552
Age (years)		59.54 (11.02)	60.37 (14.25)	0.7627
Height (cm)		163.57 (6.18)	163.95 (6.86)	0.783
BMI (kg/m^2^)		23.12 (3.26)	23.72 (3.10)	0.3866
Weight (kg)		61.89 (9.64)	63.57 (7.89)	0.3793
ASA classification (n, %)	I	5 (10.87)	4 (9.76)	0.0952
	II	41 (89.13)	33 (80.49)	0.1252
	III	0 (0.00)	4 (9.76)	0.0852
Single lung ventilation time (min)		57.50 (35.00, 90.00)	45.00 (35.00, 82.00)	0.4924
Intraoperative bleeding (ml)		25.00 (20.00, 25.00)	25.00 (20.00, 25.00)	0.575
Operative side (%)	left	24 (52.17)	13 (31.71)	0.0872
	right	22 (47.83)	28 (68.29)	
Propofol (mg)		415.00 (292.5, 586.0)	482.00 (297.0, 592.0)	0.586
Cisatracurium (mg)		22.00 (21.0, 25.0)	22.00 (18.0, 26.0)	0.531
Sufentanil (µg)		42.00 (35.0, 47.0)	35.00 (30.5, 45.5)	0.100
Remifentanil (µg)		153.50 (125.0, 204.0)	152.00 (116.0, 203.5)	0.250
Sevoflurane (ml)		22.50 (15.0, 30.0)	30.00 (20.0, 30.0)	0.439

*Abbreviations* *BMI * Body Mass Index, *ASA * American Society of Anesthesiologists, *% *percentage(s), *m * meter(s), *kg * kilogram(s), *cm * centimeter(s), *min * minute(s), *ml * milliliter, *mg * milligram(s), *µg * microgram(s)

### Postoperative pain

**Fig. 4:**
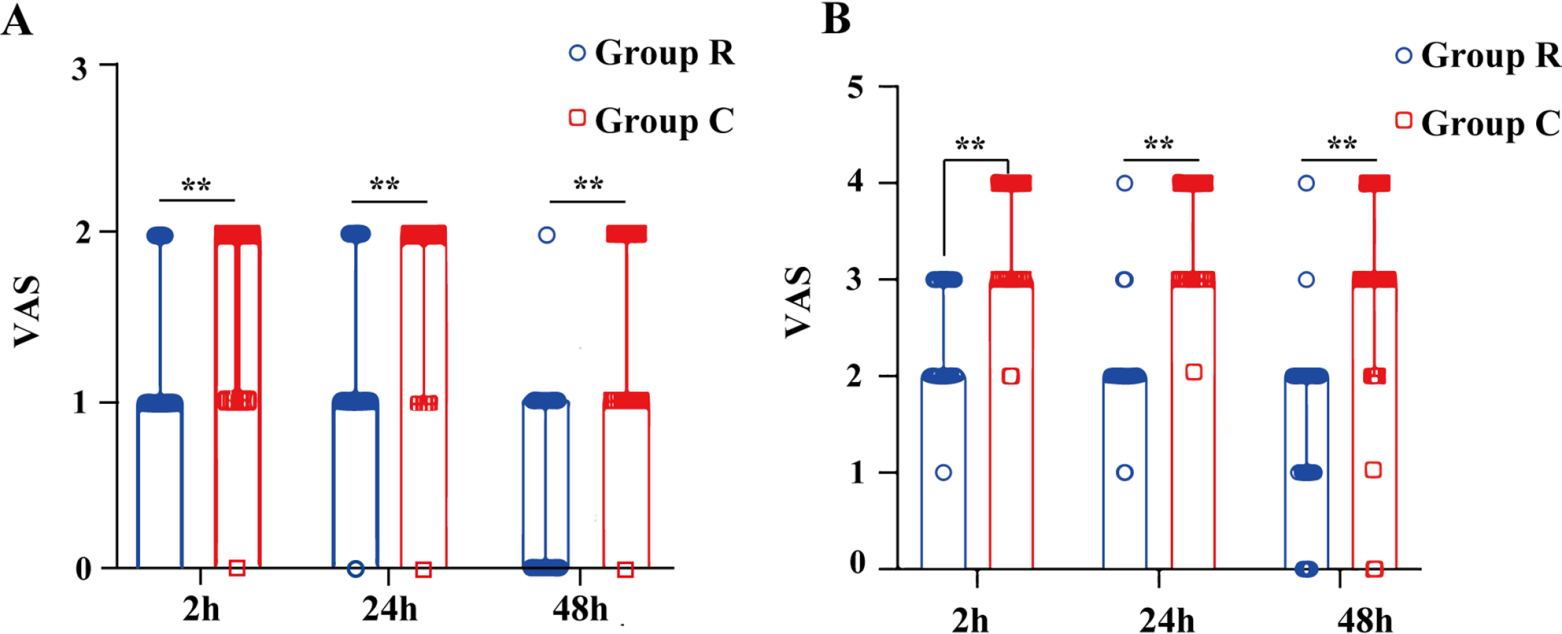
Comparison of postoperative VAS score-time curves and sufentanil consumption. **A** Curves of resting VAS scores over time in the two groups; **B** Curves of movement VAS scores over time in the two groups

**Fig. 5:**
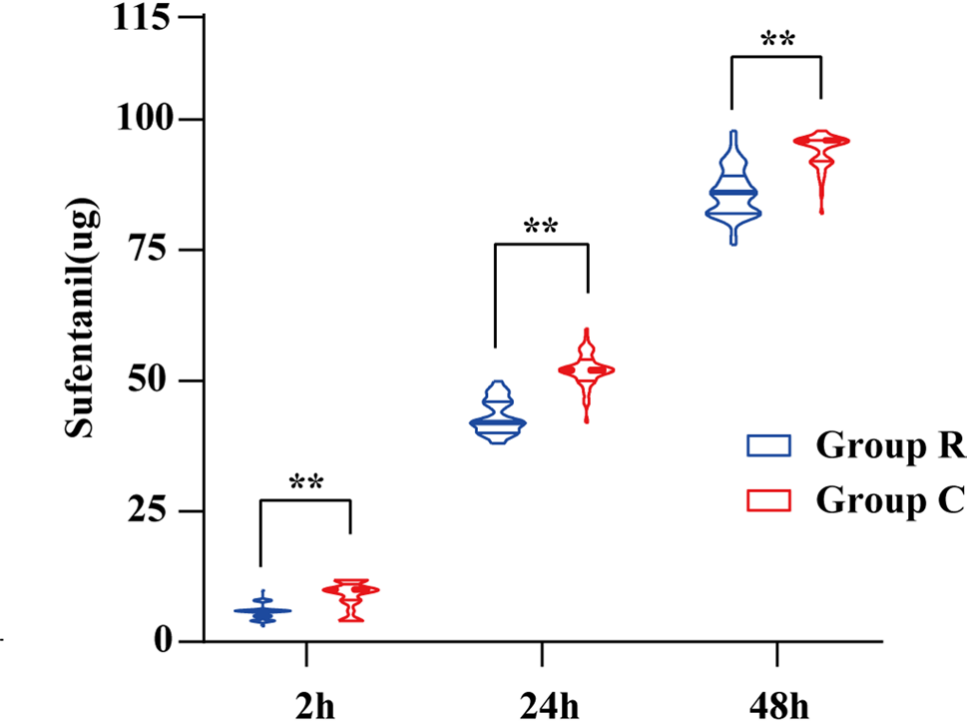
Postoperative sufentanil consumption

The VAS scores of patients in Group R at rest and during movement at 2 h, 24 h, and 48 h after surgery were all lower than those in Group C, and the differences were statistically significant (*P* < 0.05). Through repeated-measures analysis of variance with Bonferroni correction, it was found that the VAS scores of Group C at rest and during movement were significantly higher than those of Group R (*P* < 0.0001), and Group C was higher than Group R in different periods. Importantly, the median intergroup differences in VAS scores exceeded 1.0 point across all assessments, surpassing the minimal clinically important difference (MCID) for postoperative pain, which has been reported to range from 1.0 to 1.3 on a 10-point scale [16–18]. This indicates that the analgesic advantage achieved with the RISS block was not only statistically significant but also clinically meaningful. (*P* < 0.0001, Table 2; Fig. 4**)**. The consumption of sufentanil in PCIA of patients in Group R at 2 h, 24 h, and 48 h after surgery was significantly less than that in Group C; the difference was statistically significant (*P* < 0.05, Fig. 5**)**.

**Table 2:** Comparison of postoperative VAS scores between the two groups

	Group R(*n* = 46) M(Q1, Q3)	Group C(*n* = 41) M(Q1, Q3)	*P* value
Resting VAS score (2 h postoperatively)	1.00(1.00, 1.00)^**^	2.00(2.00, 2.00)	< 0.0001
Resting VAS score (24 h postoperatively)	1.00(1.00, 1.00)^**^	2.00(2.00, 2.00)	< 0.0001
Resting VAS score (48 h postoperatively)	0.00(0.00, 1.00)^**^	1.00(1.00, 2.00)	< 0.0001
Movement VAS score (2 h postoperatively)	2.00(2.00, 3.00)^**^	3.00(3.00, 4.00)	< 0.0001
Movement VAS score (24 h postoperatively)	2.00(2.00, 2.00)^**^	3.00(3.00, 4.00)	< 0.0001
Movement VAS score (48 h postoperatively)	2.00(1.00, 2.00)^**^	3.00(2.00, 4.00)	< 0.0001

Note: Compared with Group C, *P* < 0.01^**^(after Bonferroni correction)

### Postoperative adverse reactions and satisfaction

There was no statistically significant difference in the adverse reactions of PONV between the two groups after the operation (*P* > 0.05); dizziness and somnolence in Group R were significantly less than those in Group C, and the difference was statistically significant (*P* < 0.05), no puncture site infection or hematoma was observed in patients in Group R; the satisfaction in Group R was significantly better than that in Group C, and the difference was statistically significant (*P* < 0.05, Table 3).

**Table 3:** Comparison of postoperative complications and satisfaction

		Group R (*n* = 46)	Group C (*n* = 41)	*P*-value
PONV(n, %)		5 (10.87)	9 (21.95)	0.2662
Dizziness(n, %)		1 (2.17) ^**^	10 (24.39)	0.0024
Somnolence(n, %)		0 (0.00) ^**^	11 (26.83)	0.0001
Puncture site infection (n, %)		0 (0.00)	0 (0.00)	1.000
Post-puncture haematoma (n, %)		0 (0.00)	0 (0.00)	1.000
Satisfaction (n, %)^#^	0	1 (2.17)	5 (12.20)	< 0.0001
	1	5 (10.87)	16 (39.02)	
	2	13 (28.26)	15 (36.59)	
	3	27 (58.70)	5 (12.20)	

*Abbreviations: PONV* postoperative nausea and vomiting; %=percentage(s)

*M* median; Q1, Q3: first and third quartiles; *VAS* visual analogue scale

### Comparison of DE between the two groups

Before the operation, there was no significant difference in DE during quiet breathing and deep breathing on the left and right sides between the two groups (*P* > 0.05); at 30 min after extubation, 2 h and 24 h after the operation, the amplitudes of DE during quiet breathing and deep breathing on the left and right sides in Group R were greater than those in Group C (*P* < 0.05). At 48 h after the operation, the DE during deep breathing on the left and right sides and during quiet breathing on the left side in Group R were greater than those in Group C (*P* < 0.05), but there was no significant difference in DE on the right side during quiet breathing between the two groups (*P* > 0.05, Fig. S1 A-D).

According to the different surgical sides of patients, this study further carried out subgroup analysis: It was found that in patients with right-lung surgery (*n* = 50), at 30 min after extubation and 2 h after the operation, the amplitudes of DE during quiet breathing and deep breathing on the left and right sides in Group R were greater than those in Group C (*P* < 0.05); at 24 h after the operation, the DE during deep breathing on the left and right sides and quiet breathing on the left side in Group R were greater than those in Group C (*P* < 0.05), but there was no significant difference in DE on the right side during quiet breathing between the two groups; at 48 h after the operation, the amplitudes of DE during deep breathing on the left and right sides in Group R were greater than those in Group C (*P* < 0.05), but there was no significant difference in DE on both sides during quiet breathing between the two groups (*P* > 0.05, Fig. S1 E-H).

It was found that in patients with left-lung surgery (*n* = 37), at 30 min after extubation, 2 h and 24 h after the operation, the amplitudes of DE during quiet breathing and deep breathing on the left and right sides in Group R were greater than those in Group C (*P* < 0.05); at 48 h after the operation, the DE during deep breathing on the left and right sides and quiet breathing on the left side in Group R were greater than those in Group C (*P* < 0.05), but there was no significant difference in DE on the right side during quiet breathing between the two groups (Fig. S1 I-L).

## Discussion

This study suggests that RISS block can modestly reduce postoperative pain of patients undergoing thoracoscopic lung cancer surgery. In this study, the combination of RISS block and PCIA can significantly reduce the VAS scores of patients at 2 h, 24 h, and 48 h after surgery. The analgesic control during turning over and sitting up was also satisfactory at each postoperative time point, supporting the analgesic benefits of the RISS block. Judging from the changing trend of VAS, Group R maintained relatively lower resting and movement pain levels at 24 h and 48 h compared with Group C, indicating that the multi-modal analgesia of RISS block combined with PCIA can effectively relieve the postoperative pain of patients for a long time, exert a more complete analgesic effect, reduce the postoperative pain degree of lung cancer surgery patients, and improve the postoperative comfort of patients. Consistent with previous studies, RISS block provided superior postoperative analgesia compared with general anesthesia alone. Reductions in both resting and movement VAS scores were statistically significant at all time points and exceeded the MCID, suggesting clinically meaningful analgesic benefits [16–18]. This reduction facilitates effective coughing and deep breathing, decreases the risk of atelectasis and pulmonary complications, and promotes early mobilization, thereby enhancing postoperative recovery. This study speculates that the main reasons for the good analgesic effect of RISS block are as follows: (1) RISS block is a combined block, that is, it combines RIB with low-level SAPB, which can block the cutaneous branches of the thoracic wall nerves from T_2_ -T_12_, to make up for the application limitations of the narrow range of simple RIB or SAPB that cannot cover all surgical incisions in thoracoscopic surgery. A randomized controlled study by Deng et al. showed that RISS block exhibits a more long-lasting analgesic effect than RIB and can obtain a lower numeric rating scale (NRS) score and a longer time to the first postoperative analgesic rescue. The reason may be that the analgesia on the skin of the chest and upper abdomen by the RISS block can be repeatedly covered [19]. (2) RISS block can achieve a wide and effective regional nerve block, block the transmission of surgical wound pain stimuli, relieve pain [20], inhibit postoperative stress and inflammation generation [21], reduce postoperative oxidative stress [22], and inhibit the increase of plasma inflammatory factors [23]. Meanwhile, the anti-inflammatory effect of RISS block can reduce central sensitization and change the excitability of pain-center neurons to relieve pain [24]. Although most surgical injuries are in the range of peripheral nerves, the inflammation and pathological changes caused by surgery may change the peripheral and central nervous systems, leading to central sensitization and postoperative chronic pain, and regional nerve block can prevent C-fiber-transmitted nociceptive information and reduce central sensitization [25]. (3) Nerve block interrupts the vicious cycle of pain. Especially when the patient is at the end of the operation, during the recovery period when opioid drugs are gradually metabolized and decreased, during extubation with severe coughing, during posture changes and getting out of bed, etc., the stimulation caused by the relative movement of the thoracic drainage tube may lead to the patient’s pain. At this time, the analgesic effect of regional nerve block is manifested. Nerve block not only blocks the local nerve conduction pathway but also cuts off the vicious cycle of increased local inflammation and pain mediators caused by muscle tension and small-vessel smooth-muscle spasm-induced pain exacerbation [26], providing double guarantees for long-term good analgesia in the follow-up. Meanwhile, the result that PCIA + RISS block can significantly reduce the sufentanil dosage after VATS in this study is consistent with the research results of Deng et al. [19].

It is noteworthy that the timely recovery of diaphragmatic function is extremely important for thoracic surgery. Previous studies generally believed that thoracic surgery can cause diaphragmatic function impairment, which may lead to diaphragmatic dysfunction (DD) and thus affect the recovery of patients’ postoperative respiratory function [27, 28]. Multiple studies have shown that perioperative DD significantly reduces lung vital capacity and pulmonary compliance, and is closely associated with postoperative pulmonary complications [29, 30]. DE is a dynamic indicator reflecting diaphragmatic contractility and respiratory mechanics [31, 32]. Based on this criterion, the incidence of DD after elective VATS for lung cancer resection may reach up to 55%, and compared with patients without DD 24 h after surgery, the incidence of postoperative atelectasis is significantly increased (35% vs. 13%) [33]. Therefore, DE examination is of critical clinical importance for identifying perioperative diaphragmatic paralysis, DD, respiratory insufficiency, and anticipating postoperative pulmonary complications.

In this study, bilateral DE measured at 30 min post-extubation and at 2 h, 24 h, and 48 h postoperatively was consistently higher in Group R, indicating that patients in the Group R experienced less diaphragmatic impairment, faster recovery of muscle strength, and greater improvements in breathing depth and duration, thereby facilitating overall respiratory functional recovery [34]. These findings suggest that the RISS block, as a regional analgesic technique, may provide potential advantages in preserving diaphragmatic function and mitigating surgical impact on respiratory mechanics. The underlying mechanisms may include: (1) The RISS block reaches the range of blocking the cutaneous branches of the thoracic wall nerves from T_2_-T_12_, which can provide a good analgesic effect for the anterolateral and posterolateral walls of the chest, thereby reducing pain-induced spasm and abnormal activity of the diaphragm and accessory respiratory muscles (e.g., intercostal and serratus anterior muscles). This facilitates deeper breathing, effective coughing, and sputum clearance, reducing the risks of atelectasis and secretion retention, and promoting early restoration of physiological respiratory patterns; (2) The RISS block planes are the rhomboid-intercostal plane and the low-level serratus anterior-intercostal plane. Compared with the commonly used SAPB, the injection plane is deeper, which avoids blockade of the long thoracic nerve [35]. Thereby preserving the function of the serratus anterior and other accessory respiratory muscles. This preservation allows these muscle groups to participate in coordinated thoracic movement during the early postoperative period, indirectly supporting diaphragmatic motion and maintaining respiratory mechanic balance.

To further exclude potential confounding factors such as phrenic nerve anatomical distribution, cardiac pulsation, and lobar anatomical differences on DE measurements, we performed a subgroup analysis stratified by the surgical side [36]. The results consistently demonstrated that, regardless of whether patients underwent right or left lung surgery, the decline in ipsilateral DE was less pronounced and recovery was faster in the Group R compared with the Group C. More notably, contralateral DE was also better preserved in the Group R than in the Group C, suggesting that the RISS block exerts a protective effect on bilateral diaphragmatic function.This may be attributable to the diaphragm functioning as an integrated unit, in which recovery trends demonstrate bilateral synchrony unless unilateral neural or muscular injury occurs [37]. From the perspective of clinical functional recovery, early improvement in DE not only directly reflects the restoration of diaphragmatic contractility but is also closely associated with postoperative recovery of lung volumes, enhanced exercise tolerance, and reduced incidence of complications. Greater diaphragmatic excursion indicates more effective ventilation efficiency and coughing ability, which in turn influence the trajectory of postoperative recovery and hospital length of stay. Therefore, by promoting the preservation and recovery of diaphragmatic function, the RISS block may play a beneficial role in accelerating postoperative rehabilitation and improving short-term patient outcomes.

This study has several limitations. Firstly, the RISS block was conducted after the operation, which hampers the detection of local anesthesia-induced complications. To maximize patient safety, all procedures were carried out by the same experienced anesthesiologist in nerve block. The water-separation technique was employed to confirm the correct block site before local anesthetic injection, ensuring block safety and efficacy. Secondly, due to ethical and safety concerns, a sham nerve block was not performed in the control group, which prevented complete double-blinding. However, standardized anesthesia protocols, blinding of outcome assessors, and the inclusion of objective outcome measures (e.g., opioid consumption and DE) were implemented to minimize the potential influence of performance and detection bias. Thirdly, the relatively small sample size and incomplete observation indicators may lead to sample selection bias. Fourthly, the short study period calls for long-term follow-up to understand the influence of ultrasound-guided RISS block on patients’ postoperative life and long-term recovery. Fifthly, with a single-center design, the conclusions require verification by multicenter clinical studies. Finally, only using ultrasound-guided RISS block as a peri-operative analgesia method, future studies should compare its effect with other regional block techniques to offer better clinical block methods.

## Conclusion

Ultrasound-guided RISS block can modestly reduce postoperative pain in patients undergoing VATS, with clinically meaningful benefits. It may help allivate diaphragmatic dysfunction caused by surgical or anesthetic factors, thereby enhancing patient comfort and promoting accelerated postoperative recovery.

## Supplementary Information

Supplementary Material 1.

## Data Availability

The data are available from the corresponding author upon reasonable request.

## Abbreviations

VATS: Video-assisted thoracic surgery
RISS: Rhomboid-intercostal and sub-serratus plane
DE: Diaphragm excursion
PCIA: patient-controlled intravenous analgesia
VAS: visual analogue scale
SAPB: Serratus anterior plane block
RIB: Rhomboid intercostal block
BIS: Bispectral index
ASA: American Society of Anesthesiologists
BMI: body mass index
NRS: numeric rating scale
DD: Diaphragmatic dysfunction

## References

[1] Landreneau RJ, Wiechmann RJ, Hazelrigg SR, Mack MJ, Keenan RJ, Ferson PF. Effect of minimally invasive thoracic surgical approaches on acute and chronic postoperative pain. Chest Surg Clin Noth Am. 1998;8(4):891–906. 9917931

[2] Hong JM, Kim E, Jeon S, Lee D, Baik J, Cho AR, et al. A prospective double-blinded randomized control trial comparing erector spinae plane block to thoracic epidural analgesia for postoperative pain in video-assisted thoracic surgery. Saudi Med J. 2023;44(2):155–63. 10.15537/smj.2023.44.2.20220644. 36773983 10.15537/smj.2023.44.2.20220644 PMC9987706

[3] Elsharkawy H, Saifullah T, Kolli S, Drake R. Rhomboid intercostal block. Anaesthesia. 2016;71(7):856–7. 10.1111/anae.13498. 27291611 10.1111/anae.13498

[4] Jonsson M, Ahlsson A, Hurtig-Wennlöf A, Vidlund M, Cao Y, Westerdahl E. In-Hospital physiotherapy and physical recovery 3 months after lung cancer surgery: A randomized controlled trial. Integr Cancer Ther. 2019;18:1534735419876346. 10.1177/1534735419876346. 31530046 10.1177/1534735419876346 PMC6751530

[5] Scott S, Fuld JP, Carter R, McEntegart M, MacFarlane NG. Diaphragm ultrasonography as an alternative to whole-body plethysmography in pulmonary function testing. J Ultrasound Medicine: Official J Am Inst Ultrasound Med. 2006;25(2):225–32. 10.7863/jum.2006.25.2.225. 10.7863/jum.2006.25.2.225 16439786

[6] Kocjan J, Gzik-Zroska B, Nowakowska-Lipiec K, Burkacki M, Suchoń S, Michnik R, et al. Thoracic surgery May alter body static balance via diaphragm dysfunction. PLoS ONE. 2022;17(8):e0273641. 10.1371/journal.pone.0273641. 36044444 10.1371/journal.pone.0273641 PMC9432710

[7] Ventura L, Zhao W, Chen T, Wang Z, Feng J, Gu Z, et al. Significant diaphragm elevation suggestive of phrenic nerve injury after thoracoscopic lobectomy for lung cancer: an underestimated problem. Translational Lung Cancer Res. 2020;9(5):1822–31. 10.21037/tlcr-20-540. 10.21037/tlcr-20-540 PMC7653126 33209604

[8] Petrof BJ, Hussain SN. Ventilator-induced diaphragmatic dysfunction: what have we learned? Current opinion in critical care. 2016;22(1):67–72.10.1097/mcc.0000000000000272 10.1097/MCC.0000000000000272 26627540

[9] Charlesworth M, Glossop AJ. Strategies for the prevention of postoperative pulmonary complications. Anaesthesia. 2018;73(8):923–27. 10.1111/anae.14288. 29582408 10.1111/anae.14288

[10] Laghi F, Tobin MJ. Disorders of the respiratory muscles. Am J Respir Crit Care Med. 2003;168(1):10–48. 10.1164/rccm.2206020. 12826594 10.1164/rccm.2206020

[11] Lerolle N, Diehl JL. Ultrasonographic evaluation of diaphragmatic function. Crit Care Med. 2011;39(12):2760–1. 10.1097/CCM.0b013e31822a55e9. 22094504 10.1097/CCM.0b013e31822a55e9

[12] Dass C, Dako F, Simpson S, Marchetti N, Steiner R, Criner G. Sonographic evaluation of diaphragmatic dysfunction: Technique, Interpretation, and clinical applications. J Thorac Imaging. 2019;34(6):W131–40. 10.1097/rti.0000000000000436. 31385877 10.1097/RTI.0000000000000436

[13] Sferrazza Papa GF, Pellegrino GM, Di Marco F, Imeri G, Brochard L, Goligher E, et al. A review of the ultrasound assessment of diaphragmatic function in clinical practice. Respir Int Rev Thorac Dis. 2016;91(5):403–11. 10.1159/000446518. 10.1159/000446518 27216909

[14] Bleustein C, Rothschild DB, Valen A, Valatis E, Schweitzer L, Jones R. Wait times, patient satisfaction scores, and the perception of care. Am J Manag Care. 2014;20(5):393–400. 25181568

[15] Bian ZX, Shang HC. CONSORT 2010 statement: updated guidelines for reporting parallel group randomized trials. Ann Intern Med. 2011;154(4):290–1. 10.7326/0003-4819-154-4-201102150-00016. author reply 91 – 2. 21320945 10.7326/0003-4819-154-4-201102150-00016

[16] Myles PS, Myles DB, Galagher W, Boyd D, Chew C, MacDonald N, et al. Measuring acute postoperative pain using the visual analog scale: the minimal clinically important difference and patient acceptable symptom state. Br J Anaesth. 2017;118(3):424–29. 10.1093/bja/aew466. 28186223 10.1093/bja/aew466

[17] Kelly AM. The minimum clinically significant difference in visual analogue scale pain score does not differ with severity of pain. Emerg Med J. 2001;18(3):205–7. 10.1136/emj.18.3.205. 11354213 10.1136/emj.18.3.205 PMC1725574

[18] Todd KH, Funk KG, Funk JP, Bonacci R. Clinical significance of reported changes in pain severity. Ann Emerg Med. 1996;27(4):485–9. 10.1016/s0196-0644(96)70238-x. 8604867 10.1016/s0196-0644(96)70238-x

[19] Deng W, Hou XM, Zhou XY, Zhou QH. Rhomboid intercostal block combined with sub-serratus plane block versus rhomboid intercostal block for postoperative analgesia after video-assisted thoracoscopic surgery: a prospective randomized-controlled trial. BMC Pulm Med. 2021;21(1):68. 10.1186/s12890-021-01432-7. 33632189 10.1186/s12890-021-01432-7 PMC7908696

[20] Oksuz M, Abitagaoglu S, Kaciroglu A, Koksal C, Ozturk BY, Erel O, et al. Effects of general anaesthesia and ultrasonography-guided interscalene block on pain and oxidative stress in shoulder arthroscopy: A randomised trial. Int J Clin Pract. 2021;75(12):e14948. 10.1111/ijcp.14948. 34614288 10.1111/ijcp.14948

[21] Lohser J, Slinger P. Lung injury after One-Lung ventilation: A review of the pathophysiologic mechanisms affecting the ventilated and the collapsed lung. Anesth Analg. 2015;121(2):302–18. 10.1213/ane.0000000000000808. 26197368 10.1213/ANE.0000000000000808

[22] Licker M, Fauconnet P, Villiger Y, Tschopp JM. Acute lung injury and outcomes after thoracic surgery. Curr Opin Anaesthesiol. 2009;22(1):61–7. 10.1097/ACO.0b013e32831b466c. 19295294 10.1097/ACO.0b013e32831b466c

[23] Huang Z, Cai Y, Yang Y, Shi J, Zhao X, Mo H, et al. Effects of ultrasound-guided lumbar-sciatic nerve block and epidural anesthesia on the levels of IL-6, IL-8, TNF-α and coagulation factors in peripheral blood of elderly patients after hip arthroplasty. J Med Biochem. 2022;41(4):433–40. 10.5937/jomb0-35847. 36381074 10.5937/jomb0-35847 PMC9618344

[24] Sunder RA, Toshniwal G, Dureja GP. Ketamine as an adjuvant in sympathetic blocks for management of central sensitization following peripheral nerve injury. J Brachial Plexus Peripheral Nerve Injury. 2008;3:22. 10.1186/1749-7221-3-22. 10.1186/1749-7221-3-22 PMC2584055 18950516

[25] Evcili G, Yabalak A. Effectiveness of peripheral nerve blockage on the symptoms of both diseases in patients with fibromyalgia and chronic migraine coexistence. Revista Da Associacao Med Brasileira (1992). 2022;68(8):1064–67. 10.1590/1806-9282.20220202. 10.1590/1806-9282.20220202 PMC9574977 36000602

[26] Vadhanan P, Tripaty DK, Adinarayanan S. Physiological and Pharmacologic aspects of peripheral nerve blocks. J Anaesthesiol Clin Pharmacol. 2015;31(3):384–93. 10.4103/0970-9185.161679. 26330722 10.4103/0970-9185.161679 PMC4541190

[27] Mead J, Loring SH. Analysis of volume displacement and length changes of the diaphragm during breathing. J Appl Physiology: Respiratory Environ Exerc Physiol. 1982;53(3):750–5. 10.1152/jappl.1982.53.3.750. 10.1152/jappl.1982.53.3.750 6215387

[28] Lagier D, Zeng C, Fernandez-Bustamante A, Vidal Melo MF. Perioperative pulmonary atelectasis: part II. Clin Implications Anesthesiology. 2022;136(1):206–36. 10.1097/aln.0000000000004009. 10.1097/ALN.0000000000004009 PMC9885487 34710217

[29] Zeng C, Lagier D, Lee JW, Vidal Melo MF. Perioperative pulmonary atelectasis: part I. Biology and mechanisms. Anesthesiology. 2022;136(1):181–205. 10.1097/aln.0000000000003943. 34499087 10.1097/ALN.0000000000003943 PMC9869183

[30] Spadaro S, Grasso S, Dres M, Fogagnolo A, Dalla Corte F, Tamburini N, et al. Point of care ultrasound to identify diaphragmatic dysfunction after thoracic surgery. Anesthesiology. 2019;131(2):266–78. 10.1097/aln.0000000000002774. 31166236 10.1097/ALN.0000000000002774

[31] Lee JH, Kang P, Park JB, Kim JT. Changes in diaphragmatic ultrasonography findings and their association with postoperative complications in children undergoing pulmonary resection: A single-centre, prospective, observational study. Eur J Anaesthesiol. 2023;40(12):953–56. 10.1097/eja.0000000000001910. 37823729 10.1097/EJA.0000000000001910

[32] Haaksma ME, Smit JM, Boussuges A, Demoule A, Dres M, Ferrari G, et al. EXpert consensus on diaphragm ultrasonography in the critically ill (EXODUS): a Delphi consensus statement on the measurement of diaphragm ultrasound-derived parameters in a critical care setting. Crit Care (London England). 2022;26(1):99. 10.1186/s13054-022-03975-5. 10.1186/s13054-022-03975-5 PMC8991486 35395861

[33] Boussuges A, Brégeon F, Blanc P, Gil JM, Poirette L. Characteristics of the paralysed diaphragm studied by M-mode ultrasonography. Clin Physiol Funct Imaging. 2019;39(2):143–49. 10.1111/cpf.12549. 30325572 10.1111/cpf.12549

[34] Valette X, Seguin A, Daubin C, Brunet J, Sauneuf B, Terzi N, et al. Diaphragmatic dysfunction at admission in intensive care unit: the value of diaphragmatic ultrasonography. Intensive Care Med. 2015;41(3):557–9. 10.1007/s00134-014-3636-6. 25600191 10.1007/s00134-014-3636-6

[35] Shang LH, Xiao ZN, Zhao YL, Long B. Analgesic effect of serratus anterior plane block after thoracoscopic surgery: A randomized controlled Double-Blinded Study. Therapeutics and clinical risk management. 2020;16:1257–6510.2147/tcrm.S285244 10.2147/TCRM.S285244 PMC7755330 33376335

[36] Kokatnur L, Rudrappa M, Basel. Switzerland). 2018;6(1). 10.3390/diseases6010016. 10.3390/diseases6010016 PMC5871962 29438332

[37] Sanchez de Toledo J, Munoz R, Landsittel D, Shiderly D, Yoshida M, Komarlu R, et al. Diagnosis of abnormal diaphragm motion after cardiothoracic surgery: ultrasound performed by a cardiac intensivist vs. fluoroscopy. Congenit Heart Dis. 2010;5(6):565–72. 10.1111/j.1747-0803.2010.00431.x. 21106016 10.1111/j.1747-0803.2010.00431.x

