# Supplementary material for: Effects of rhomboid intercostal and sub-serratus plane block on perioperative analgesic efficacy and diaphragm excursion in video-assisted thoracic surgery: a prospective, randomized controlled trial
Source: BMC Anesthesiol. 2025 Dec 18;26:50. doi: 10.1186/s12871-025-03556-3 (PMC12828999; doi:10.1186/s12871-025-03556-3)
Supplement: Supplementary file 1 — Supplementary Material 1. [file 12871_2025_3556_MOESM1_ESM.docx]

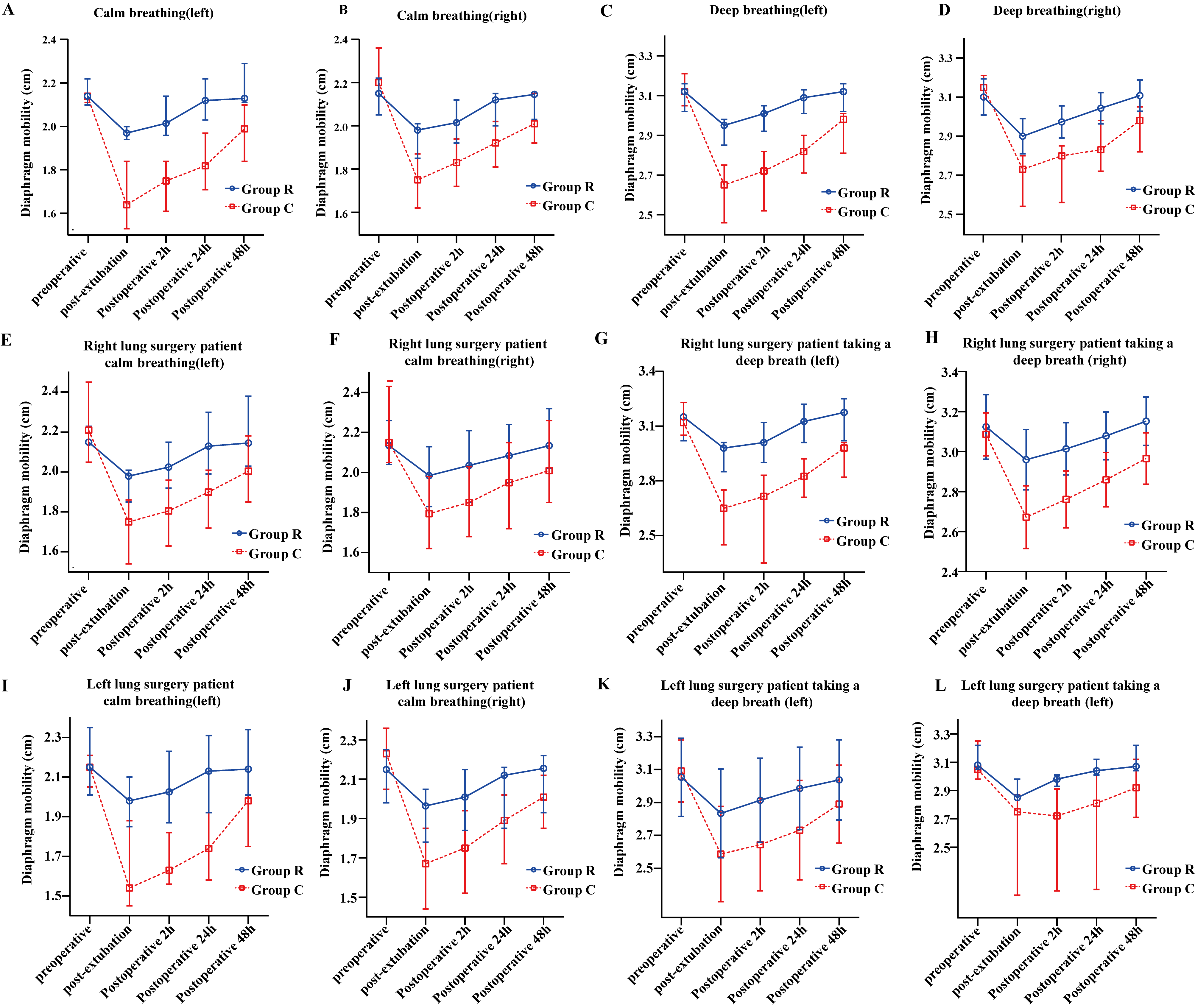


**Fig. S1 Comparison of DE in patients of the two groups and patients of right-lung and left-lung surgeries.**

A. DE of left side calm breathing in two groups of patients(cm); B. DE of right-side calm breathing in two groups of patients (cm); C. DE of deep breathing on the left side of patients in two groups (cm); D. DE of calm breathing on the right side of patients in two groups (cm); E. DE of calm breathing on the left side of patients in two groups for right lung surgery (cm); F. DE of calm breathing on the right side of patients in two groups of right lung surgery (cm); G. DE of deep breathing on the left side of patients in two groups of right lung surgery (cm); H. DE of deep breathing on the right side in two groups of patients undergoing right lung surgery (cm); I. DE of calm breathing on the left side in two groups of patients undergoing left lung surgery (cm); J. DE of calm breathing on the right side in two groups of patients undergoing left lung surgery (cm); K. DE of deep breathing on the left side in two groups of patients undergoing left lung surgery (cm); L. DE of deep breathing on the right side in two groups of patients undergoing left lung surgery (cm)
